# Machine learning models for clinical and structural knee osteoarthritis prediction: Recent advancements and future directions

**DOI:** 10.1016/j.ocarto.2025.100654

**Published:** 2025-07-24

**Authors:** Gabby B. Joseph, Charles E. McCulloch, Michael C. Nevitt, Nancy E. Lane, Sharmila Majumdar, Thomas M. Link

**Affiliations:** a Department of Radiology and Biomedical Imaging, University of California, San Francisco, USA; b Department of Epidemiology and Biostatistics, University of California, San Francisco, USA; c Department of Rheumatology, University of California, Davis, USA

**Keywords:** Machine learning, Osteoarthritis, Prediction modeling, Pain

## Abstract

Machine learning (ML), increasingly used for predictive modeling, has seen rapid growth in osteoarthritis (OA) research over the past decade. This review highlights recent advances in ML model development across four OA outcome domains: clinical, structural (radiographic and MRI-based), and surgical endpoints, each addressing different but interrelated aspects of the disease.

For clinical outcomes, ML studies have focused on predicting changes in patient-reported clinical measures (e.g., pain and function). Radiographic OA has been characterized using deep learning (DL) models, and ML approaches have also been used to predict progression of Kellgren Lawrence grades and joint space narrowing. For MRI-based features, DL-based tools have been developed for automatic quantification of cartilage, bone marrow lesions, and subcutaneous fat; they have improved scalability and supported development of ML prediction models with cartilage loss outcomes. For total knee replacement outcomes, ML models have demonstrated strong performance, offering the potential for both early intervention and surgical planning.

This review also discusses emerging directions for ML in OA research, including the integration of multimodal data sources, the development of interpretable and explainable ML models, and the use of automated ML to streamline model development. Future approaches may include OA subtype-specific prediction models, alignment of ML approaches with clinical workflows, and enhanced external validation to ensure generalizability. These evolving strategies underscore the growing potential of ML to improve the detection of early OA, individualized risk stratification, and personalized interventions in OA clinical care.

## 1 Introduction

Osteoarthritis (OA) is a heterogeneous disease that affects approximately 32.5 million adults in the United States [1] and 528 million people globally [2]. Symptoms include pain, stiffness after inactivity, tenderness, reduced flexibility, and swelling [3]. OA of the knee, which is the most commonly affected joint [4], is characterized by a complex interaction and degeneration of tissues, including cartilage, menisci, synovium, ligaments, subchondral bone, and bone marrow. Additionally, biomechanical factors such as joint malalignment, previous injury, and obesity can accelerate disease progression [5]. Given the multi-factorial nature of OA progression, there are currently no effective treatments to prevent the disease [6], and severe cases often result in total knee replacement surgery. The direct medical cost of OA in the United States is estimated at $72 billion, based on average cost data from 2008 to 2011 [2]. Thus, the high prevalence of OA and associated treatment costs highlight the need for the development of effective early treatment strategies.

Developing prediction tools for OA would enable the identification of individuals who are likely to develop OA and those in the early stages likely to progress to more advanced disease, informing targeted, individualized prevention and treatment strategies. Early identification of patients at risk for progression would enable preventive measures to be implemented during the initial stages of the disease, potentially mitigating irreversible joint damage. Individualized prediction models could help identify patients with modifiable risk factors, enabling targeted interventions and improving the selection of participants for clinical trials. Individualized models are especially important given the diverse OA phenotypes that have been identified, such as inflammatory, hypertrophic, bone marrow, and meniscal [7,8], which may have different rates of progression and distinct risk factors. Furthermore, with the development of weight loss drugs, such as GLP-1 receptor agonists, ML models offer the potential to predict which patients will likely benefit from these interventions and the extent of weight loss achievable for each individual. Since knee OA progresses at different rates among individuals, some experiencing rapid deterioration while others decline more gradually, predictive models that identify those with rapid progression could aid drug development by eliminating the recruitment of individuals with slower or minimal disease progression. Therefore, prediction tools have the potential to advance personalized treatment strategies for OA by focusing targeted interventions on individuals at high risk of progression.

Machine learning (ML), increasingly used for predictive modeling, has seen rapid growth in OA research with a notable surge in recent years [9]. ML models analyze complex datasets to assess whether predictors, such as demographic factors, MRI measurements, and biochemical markers, can predict late-stage outcomes such as total knee replacement (TKR) or radiographic progression. By modeling complex interrelationships between predictors, ML models can identify patterns and interrelationships that may not be apparent through traditional statistical methods. ML methods are particularly useful in OA prediction tasks involving high-dimensional data, non-linear relationships, feature interactions, or unstructured inputs. Unlike traditional statistical models, ML approaches offer greater flexibility and are often better suited for optimizing predictive performance in complex datasets. Early studies have utilized methods such as best subsets selection for model building [10], while more recent approaches have incorporated techniques including LASSO and Elastic Net (for feature selection), random forest, gradient boosting machines, and XGBoost (for handling complex interactions and non-linear relationships). Large OA observational study databases with rich longitudinal clinical and imaging data, such as the Osteoarthritis Initiative (OAI), the Multicenter Osteoarthritis Study (MOST), and the Rotterdam Study cohorts [[11], [12], [13]], have been instrumental in advancing this technology.

The OAI [12] is a multicenter, 8-year longitudinal study in 4796 participants at risk for OA, with data including patient-reported outcomes, radiographic and magnetic resonance (MR) images, genetics, biospecimens, and clinical outcomes, which facilitates the development and validation of prediction models. MOST [13] is a community-based cohort observational study of over 4500 older adults with or at risk for knee OA, who were monitored over 10 years. It records data on risk factors, physical activity, strength, and outcomes through radiographic and MR imaging, clinical measures, and patient-reported symptoms. The Rotterdam Study [11] (initially 7983 participants and 14,926 participants as of 2008) is a large population-based cohort of participants over 45 years that investigates chronic diseases in aging, including OA, and includes genetic data, radiographic images, MR images, and clinical outcomes. Other databases, including the Framingham Osteoarthritis Study [14], the Cohort Hip and Cohort Knee (CHECK) study [15], and the Musculoskeletal Pain in Ullensaker STudy (MUST) study [16] are also available. Together, these databases provide resources for developing robust machine learning-based prediction models and validating their performance.

This review examines advancements over the past 5 years in the application of ML models for predicting OA longitudinal outcomes. It is organized by outcome type: patient-reported clinical measures (e.g., pain and function), imaging-based measures (e.g., Kellgren-Lawrence grades from radiographs, cartilage damage and loss from MRIs), and surgical outcomes (i.e., total knee replacement). Grouping by outcome type reflects the distinct clinical goals addressed by these models, such as predicting structural progression and patient-reported outcomes, which may involve different pathophysiological pathways. This review highlights methodologies, key findings, clinical relevance, limitations, and future directions for ML applications in OA research and clinical care. We identified relevant and impactful studies through an informal search process using PubMed, applying combinations of keywords such as 'osteoarthritis,' 'machine learning,' and 'prediction.' This was not a formal systematic review, and the included studies represent a selection from the past five years that reflect major trends in the field.

## 2 Machine learning techniques and workflow

**Fig. 1:**
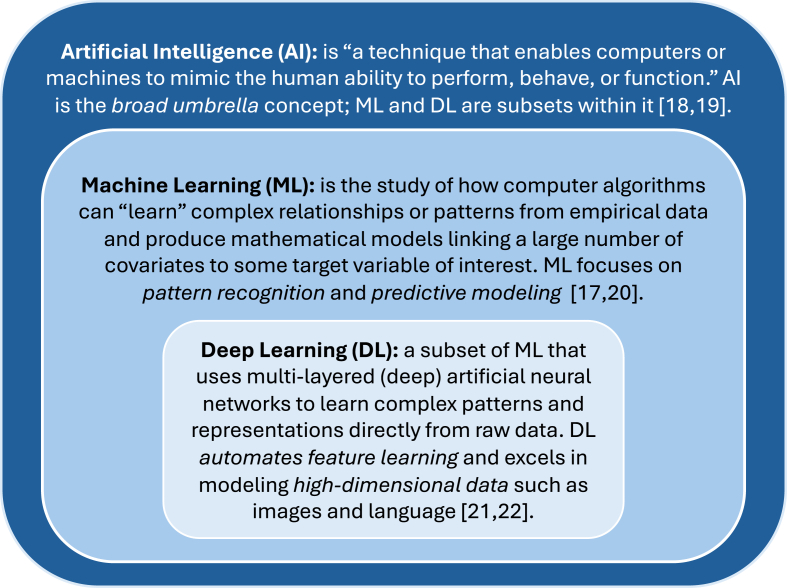
Definitions of artificial intelligence, machine learning, and deep learning

**Fig. 2:**
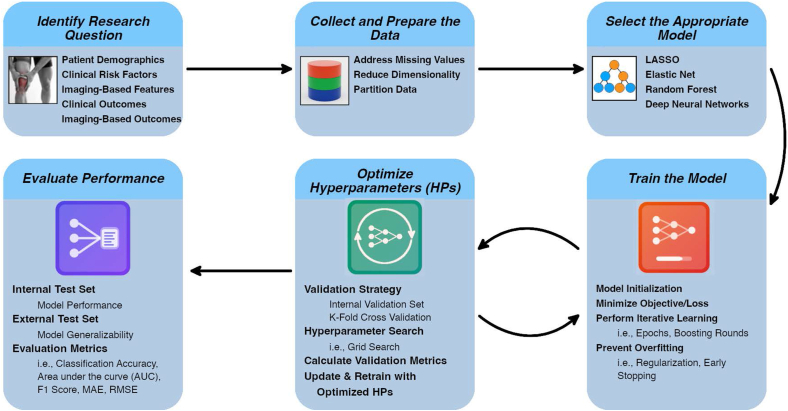
Machine Learning workflow. Key stages in Machine Learning model development: Research question identification, data collection and preparation, model selection, model training, hyperparameter optimization, and performance evaluation.

The general workflow for developing ML models is briefly outlined below. For greater depth, please refer to our previous review article (Joseph et al., 2022 [17]). The definitions of artificial intelligence (AI) [18,19], machine learning (ML) [17,20], and deep learning (DL) [21,22] are presented in Fig. 1. While this review focuses primarily on ML techniques used in OA prediction models, it also includes studies applying DL models, a subset of ML, particularly for extracting and analyzing imaging features. ML and DL studies are discussed in relation to their methodological differences and varied applications, with some studies using ML, others DL, and some incorporating both approaches. A standard ML workflow includes the following steps: identifying the research question, collecting and preparing the data, selecting an appropriate model, training the model, optimizing its parameters, and evaluating its performance (Fig. 2).

### 2.1 Identifying the research question; collecting and preparing the data

As with any scientific investigation, a research question should be clearly defined (e.g., identifying baseline patient characteristics associated with the risk of total knee replacement after 8 years). Then, data are gathered, for example, from publicly available datasets such as those previously described. Preprocessing steps include addressing missing values (e.g., using statistical or model-based imputation), normalizing features (e.g., z-scoring), and reducing dimensionality to minimize overfitting. When working across cohorts, harmonization methods may be applied to account for differences in data collection (e.g., imaging protocols). To avoid data leakage, preprocessing should be performed within each data partition. Typically, data are split into training and test sets, with some workflows including a separate validation set to tune model parameters and assess stability.

### 2.2 Selecting an appropriate model, training the model, optimizing its parameters

For model selection, various ML and DL approaches may be considered. Common ML algorithms include regularized regression methods such as LASSO and elastic net, as well as ensemble techniques such as random forest and gradient boosting machines. DL involves architectures such as convolutional neural networks (CNNs), which are well-suited for image-based tasks such as image segmentation.

Model parameters are optimized, which involves testing different combinations of settings (such as the number of trees in a random forest or the learning rate in a neural network) to identify the best-performing model. This can be done using the validation set or through k-fold cross-validation, where the training data are divided into k equally sized subsets (folds), and the model is iteratively trained and evaluated on different combinations of these folds.

### 2.3 Evaluating model performance

Model performance (evaluated on the test set) can be assessed using a variety of metrics. These may include:

• ***Classification accuracy*,** the number of correct predictions vs. the total number of input samples.
• **Area Under the Curve (AUC)**: quantifies the model's ability to discriminate between classes.
• **Sensitivity and Specificity**: measure how well the model identifies true positives and true negatives, respectively.
• **F1 Score or Dice Score**: particularly relevant for imaging applications; defined as twice the area of overlap between the predicted and true regions divided by the total number of pixels in both segmentations.
• **Mean Absolute Error (MAE):** the mean of the absolute differences between observed and predicted values.
• **Root Mean Squared Error (RMSE)**: the square root of the mean of the squared differences between observed and predicted values.
• **Discrimination**: Assesses how well the model distinguishes between individuals with and without the outcome.
• **Calibration**: Evaluates how closely predicted probabilities agree with actual outcomes.
• **Clinical Usefulness**: Assesses whether the model leads to improved decision-making in practice.

Evaluating a model on data it has not seen during training, whether using a dedicated hold-out set or employing cross-validation, is critical to ensure that its predictive performance is not overstated. To further assess generalizability (the extent to which model performance holds across different datasets or populations), models may also be evaluated on external datasets. This provides insight into whether the model performs consistently across a broader range of scenarios (i.e., populations, imaging protocols).

### 2.4 Challenges

Despite recent advances, ML models, including DL, face several challenges. These include limited generalizability, difficulty interpreting model predictions (especially with complex models such as deep neural “black box” networks that may not be explainable), and overfitting, particularly in high-dimensional datasets with relatively small sample sizes.

The following sections describe ML methods that have been used in OA applications in the past 5 years. A summary is provided in Table 1.

**Table 1:**
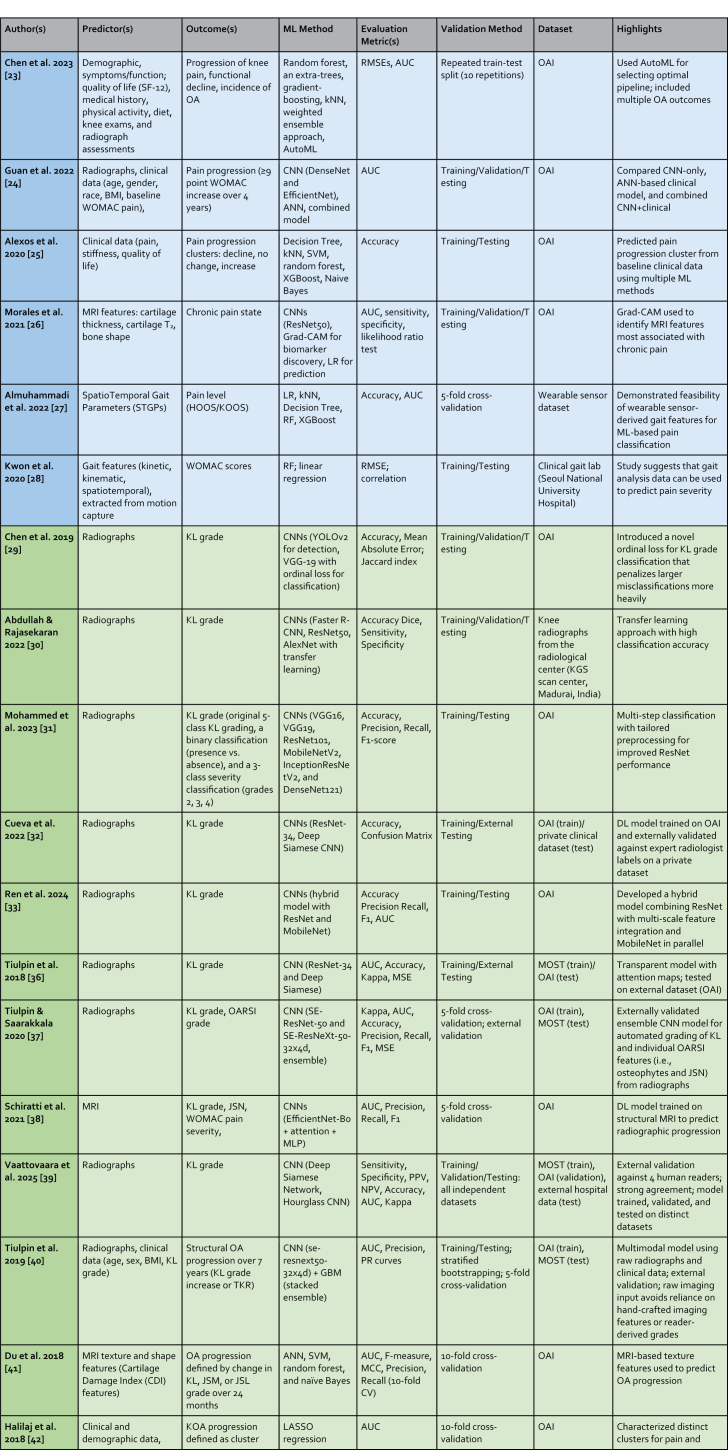

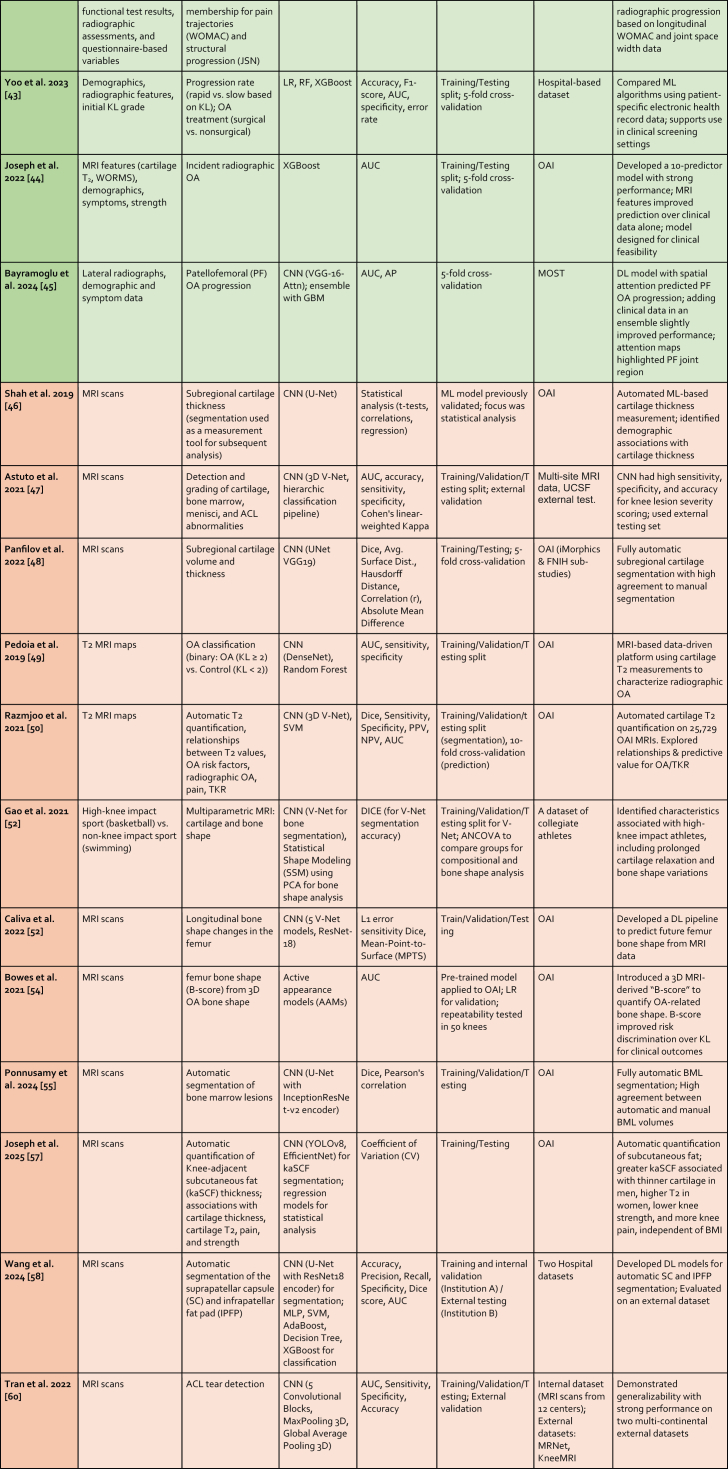

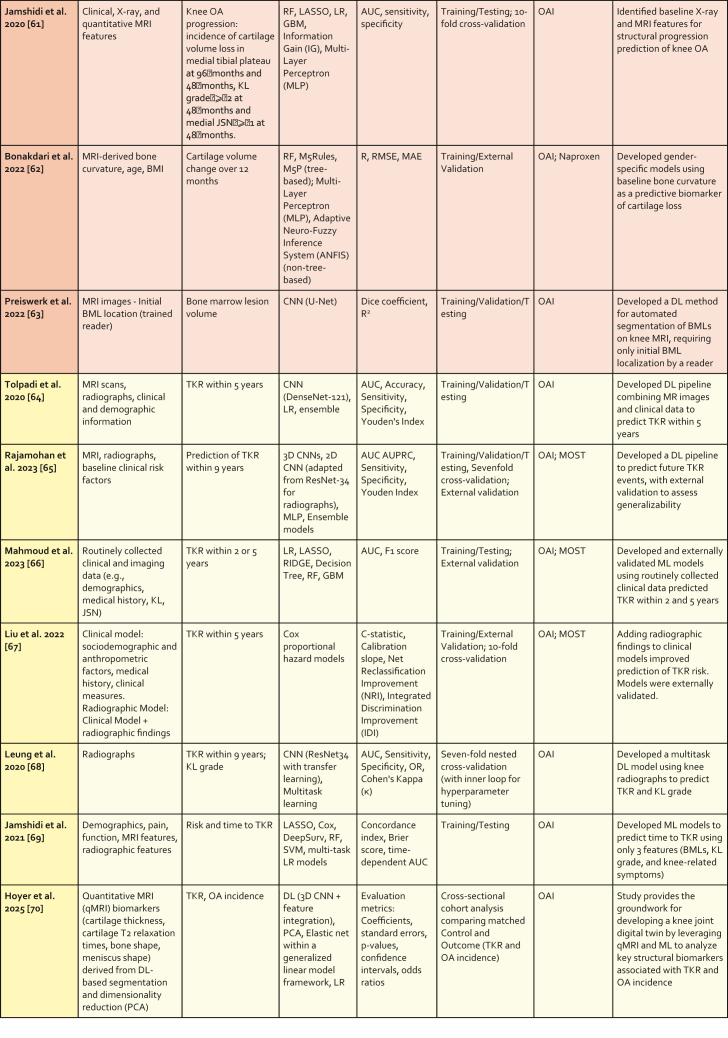
Overview of Recent Machine Learning and Deep Learning Studies in Osteoarthritis Applications. Studies are presented sequentially as discussed in the manuscript. Section color coding denotes the primary outcome measured: Blue for Symptomatic Outcomes, Green for Radiographic Outcomes, Orange for MRI Outcomes, and Yellow for TKR Outcomes.

Abbreviations: AdaBoost: Adaptive Boosting; AlexNet: (No full form commonly used, it's the name of a specific CNN architecture); ANFIS: Adaptive Neuro-Fuzzy Inference System; ANN: Artificial Neural Network; AP: Average Precision; AUC: Area Under the Curve; AUPRC: Area Under the Precision-Recall Curve; AutoML: Automated Machine Learning; Avg. Surface Dist.: Average Surface Distance; BML: Bone Marrow Lesion; C-index: Concordance Index; C-statistic: Concordance statistic; CNN: Convolutional Neural Network; CV: Coefficient of Variation; Deep Siamese CNN: Deep Siamese Convolutional Neural Network; DeepSurv: Deep Survival (analysis); DenseNet: Densely Connected Convolutional Network; Dice: Dice Similarity Coefficient; EfficientNet: Efficient Network; Faster R-CNN: Faster Region-based Convolutional Neural Network; GBM: Gradient Boosting Machine; Grad-CAM: Gradient-weighted Class Activation Mapping; IDI: Integrated Discrimination Improvement; InceptionResNetV2: Inception Residual Network version 2; JSL: Joint Space Lateral; JSM: Joint Space Medial; JSN: Joint Space Narrowing; KL: Kellgren Lawrence; kNN: k-Nearest Neighbors; LASSO: Least Absolute Shrinkage and Selection Operator; LR: Logistic Regression; MAE: Mean Absolute Error; MCC: Matthews Correlation Coefficient; MLP: Multi-Layer Perceptron; MobileNetV2: Mobile Network version 2; MOST: Multicenter Osteoarthritis Study; MSE: Mean Squared Error; NPV: Negative Predictive Value; NRI: Net Reclassification Improvement; OAI: Osteoarthritis Initiative; OR: Odds Ratio; PF: Patellofemoral; PPV: Positive Predictive Value; PCA: Principal Component Analysis; R: Correlation Coefficient (typically Pearson's R); ResNet: Residual Network; RF: Random Forest; RMSE: Root Mean Square Error; SVM: Support Vector Machine; TKR: Total Knee Replacement; U-Net: (No full form commonly used, it's the name of a specific CNN architecture); VGG-19: Visual Geometry Group - 19 layers; V-Net: (No full form commonly used, it's the name of a specific CNN architecture); XGBoost: Extreme Gradient Boosting; YOLOv2: You Only Look Once version 2.

## 3 Pain and functional outcome prediction

Pain and functional outcomes in OA are indicators of symptomatic disease progression that directly impact a patient's quality of life. ML models have been developed to predict pain progression and functional decline using outcomes including Western Ontario and McMaster Universities Osteoarthritis Index (WOMAC) subscales (i.e., pain, function) and Knee Injury and Osteoarthritis Outcome Score (KOOS) subscales. Some studies have used continuous pain outcomes [23], some have binarized pain scores [24], and others have used pain clusters as outcomes (1: pain decline, 2: no significant pain change, and 3: pain increase) based on linear regression slopes of WOMAC pain over 4 visits [25]. Integrating diverse data sources such as clinical assessments, imaging biomarkers, and patient-reported outcomes in ML models moves the field toward individualized prediction tools to facilitate personalized treatment strategies for pain reduction through early intervention. In addition, a DL data-driven approach for modeling chronic knee pain with MRI-based bone shape, cartilage thickness, and cartilage T_2_ has been described [26]. Three notable recent studies (within the last 3 years) with varied methodologies are described below:

Guan et al. (2022) [24] developed DL models to predict knee pain progression (defined as a ≥9-point increase in WOMAC pain over 48 months) using baseline data from the OAI. They evaluated three approaches: (1) a traditional model using an artificial neural network (ANN) with six baseline demographic, clinical, and radiographic variables (age, gender, race, BMI, WOMAC pain, and Kellgren-Lawrence grade); the ANN architecture matched that of prior OA risk assessment models shown to have high diagnostic performance; (2) a convolutional neural network (CNN) trained on baseline knee radiographs; and (3) a combined model with demographic, clinical, and radiographic risk factors with DL analysis of knee radiographs. The CNN outperformed the traditional model (AUC 0.77 vs. 0.69), while the combined model achieved the highest overall performance (AUC 0.81), highlighting the added value of image-based features in predicting pain progression. The performance of this study was evaluated using a hold-out dataset from the OAI, which is a strength. However, external validation on images from different radiography systems and populations is still needed to assess generalizability (as noted above).

Chen et al. (2023) [23] applied automated ML (AutoML) to OAI data (3200 high-risk individuals, 1094 clinical and imaging features) to predict 9-year trajectories of knee pain (WOMAC pain scores) and functional scores (WOMAC physical function). AutoML is a technique that streamlines the model-building process by automating key steps, including model selection, hyperparameter tuning, and optimization. The AutoML approach trained and evaluated nine ML models; of these, the weighted ensemble model (uses a multi-layer strategy that integrates multiple ML models), CatBoost model (a gradient boosting algorithm designed to handle categorical features effectively), and Extra-Trees model (a method that builds highly randomized decision trees) performed the best in predicting knee pain. Each of these models achieved a mean root mean squared error (RMSE) of 2.27 (SD ​= ​0.16) on the 0–20 WOMAC scale. The models identified baseline self-reported questionnaires (i.e., pain, function, walking speed) and some radiographic features among the top predictors. A strength of AutoML is that it systematically explores various model architectures and parameter settings, identifying high-performing models that may be less likely to be identified through manual methods (i.e., selecting algorithms and tuning hyperparameters by trial and error or relying on expert intuition), thereby reducing variability in model development and enhancing the scalability of ML applications.

Other studies have utilized gait features to predict pain in OA using ML [27,28]. Almuhammadi et al. (2022) [27] used ML to classify patients’ self-reported pain levels (HOOS/KOOS) based on SpatioTemporal Gait Parameters (STGPs) extracted from accelerometer signals from an anterior-posterior wearable sensor attached to the lumbar region during a 2-min walk at a self-selected pace. The study utilized ML models, including Decision Trees, Support Vector Machines, Random Forests, and Gradient Boosting, to classify pain severity based on gait parameters extracted from wearable sensor data. The Decision Tree model achieved 86.79 % accuracy for hip OA classification, while SVM reached 83.57 % accuracy for knee OA, demonstrating the feasibility of using ML for objective pain assessment. This study indicates that wearable sensors combined with machine learning algorithms may enable automated assessment of pain levels in individuals with hip and knee OA.

## 4 Radiographic outcomes

In the last decade, researchers have developed DL models for automatic evaluation of radiographic features that assess OA radiographic severity [[29], [30], [31], [32], [33]], with the goal of standardizing radiographic evaluations, reducing inter-reader variability, and enhancing diagnostic efficiency. DL models can extract relevant features directly from radiographs, potentially enhancing diagnostic efficiency in clinical practice. In addition, other ML approaches, such as Random Forests and Gradient Boosting Machines, have also been developed to predict future structural progression using longitudinal databases, including the OAI and MOST. Common outcome measures have included radiographic Kellgren-Lawrence (KL) [34] grading and joint space narrowing [[35], [36], [37], [38]], with predictive features from demographic, clinical, and imaging data. Four recent and notable research studies (within the last 3 years) are reviewed below:

DL-based image extraction: Vaattovaara et al. (2025) [39] utilized DL to automatically assess KL grades in the knee. The model was trained on all radiographs in the MOST study, validated using data from 3000 individuals from the OAI, and tested on an external dataset of 208 knee radiographs. The DL model demonstrated substantial agreement with the readers, achieving an overall kappa of 0.82. For multi-class KL grading (0–4), the model achieved an AUC of 0.89; Performance improved when simplified to binary classification (KL ​≥ ​2 vs. <2), with an AUC of 0.97. These findings suggest that the model can perform KL grading with diagnostic accuracy comparable to experienced clinicians, supporting its potential for clinical application. A key strength of this study is the use of an external test set, a method applied in previous studies [33,36,37], therby enhancing confidence in the model's generalizability. Nonetheless, challenges remain in ensuring robustness to uncommon radiographic features such as hardware and severe deformities; as such, a radiologist's expertise is critical.

ML-based prediction models: Research studies using ML models to predict future OA progression based on radiographic outcomes have advanced over the past decade [[40], [41], [42]]. In one of these studies, Yoo et al. (2022) [43] analyzed data from 2151 patients (screened from an initial cohort of 83,280 knees collected from a single institution's clinical data warehouse between 2003 and 2019) to evaluate whether age, sex, body mass index, bone mineral density, occupation, comorbidities, and initial KL grade could predict radiographic progression. Progression was defined as an increase in KL grade of greater than 2 from a baseline KL grade of 0–2, indicating a shift from none or early OA to more advanced disease. Among those who progressed, cases were further categorized as rapid (progression within ≤7 years) or slow (progression over >7 years). Logistic regression, random forest, and extreme gradient boosting (XGB) models were trained on patient-specific variables to predict this binary progression outcome, with XGB demonstrating the highest performance (AUC ​= ​0.68) through 5-fold cross-validation. These results support the potential of ML models, particularly XGB, to predict radiographic OA progression using accessible clinical and demographic data.

**Fig. 3:**
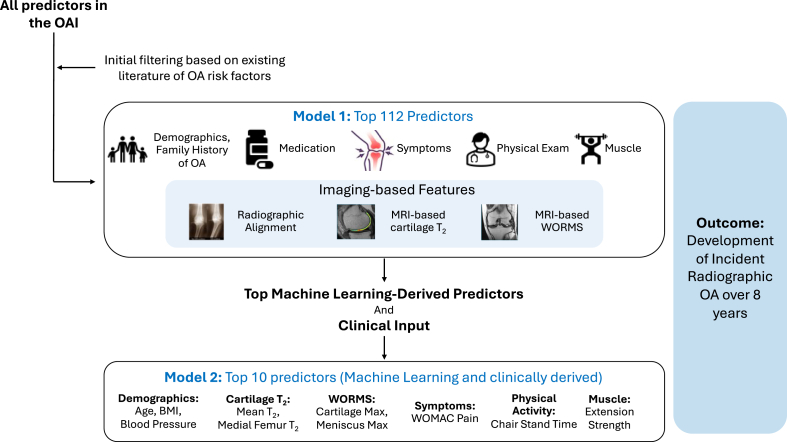
An example schema of a machine learning prediction model using OAI data based on a study by Joseph et al. [44] in 1044 individuals with KL grades 0–1 in the right knee at baseline. The outcome was defined as incident radiographic OA, characterized by progression to KL grades 2–4 over 8 years (n ​= ​183), while non-progressors remained at KL 0–1 (n ​= ​861). Model 1 included 112 predictors, including demographics, symptoms, muscle strength, physical activity, cartilage T_2_, and WORMS scores. Model 2 reduced these predictors to the top 10 based on feature importance and clinical feasibility. Model 2 achieved an AUC of 0.77 compared to 0.79 in the full model, suggesting that a more streamlined, clinically practical ML model can still retain strong predictive performance. *Abbreviations: ML, machine learning; OAI, Osteoarthritis Initiative; KL, Kellgren Lawrence; AUC, area under the receiver operating characteristic curve; WORMS, Whole-Organ Magnetic Resonance Imaging Scores; WOMAC, Western Ontario and McMaster Universities Osteoarthritis Index.*

Recent studies have increasingly incorporated MRI-based biomarkers to enhance the prediction of radiographic knee OA progression. Joseph et al. (2022) [44] developed a ML model to predict 8-year progression to radiographic OA (defined as the development of KL grade 2–4) in 1044 participants from the OAI with KL 0–1 in the right knee at baseline. The model incorporated 3T MRI-derived cartilage T_2_ values, which reflect collagen integrity and hydration status, Whole-Organ Magnetic Resonance Imaging Scores (WORMS) for semiquantitative assessment of joint structure, demographic and clinical variables, pain questionnaires, and physical activity measures. Using an XGB algorithm, the full model with 112 predictors achieved an AUC of 0.79. A reduced model with 10 clinically feasible predictors, including chair stand time, age, cartilage T_2_, WORMS scores, and BMI, demonstrated only a modest reduction in performance (AUC ​= ​0.77). In contrast, a model excluding imaging predictors performed substantially worse (AUC ​= ​0.67), highlighting the added predictive value of MRI-based features in radiographic OA risk prediction (see Fig. 3).

Most ML models predicting radiographic OA progression have focused on the tibiofemoral knee compartment; however, a recent study by Bayramoglu et al. (2024) [45] shifted focus to the patellofemoral joint. Using data from the MOST study (1832 individuals, 3276 knees), the authors applied a deep convolutional neural network (CNN) to lateral knee radiographs and clinical features, including age, sex, BMI, WOMAC score, and tibiofemoral KL grade, to predict 7-year progression of patellofemoral OA. The CNN achieved an AUC of 0.86, outperforming the XGB model based on clinical predictors alone (AUC ​= ​0.77). An ensemble model combining clinical and imaging data further improved performance (AUC ​= ​0.87). While the model was compartment-specific and not validated for overall knee OA progression, the results highlight the potential of ML to capture localized disease progression; however, the absence of a true hold-out test set may overestimate model performance and limit assessment of generalizability.

## 5 MRI-based structural outcomes

MR imaging enables the evaluation of soft tissue structures of the knee, including cartilage lesions and bone marrow edema, which are not visible on radiographs and may exhibit changes prior to detection on radiographs. Thus, MR imaging features can be useful in identifying early features of OA progression that may precede more advanced joint degeneration. In the past 5 years, DL algorithms have been applied to automate the segmentation and quantification of various structures of the knee, including cartilage thickness and focal lesions [[46], [47], [48]], cartilage T_2_ [49,50], bone shape [[51], [52], [53], [54]], bone marrow lesions [55,56], knee subcutaneous fat [57], knee synovitis [58], meniscal tears [59], and ACL tears [60]. These models show promise for improving scalability and consistency in image analysis across large datasets for both research and clinical applications. In addition, ML models for the prediction of OA progression using MR imaging outcomes have included features such as cartilage volume loss [61,62]. The following section highlights recent research studies with MR imaging-based outcomes for OA prediction.

Most recent studies using ML techniques on MR images have been focused on the application of DL techniques to segmentation and quantification of knee joint structures. Notable projects include automatic segmentation of cartilage in the entire OAI dataset, enabling quantification of cartilage thickness [46] and cartilage T_2_ [50] as well as their changes over time. Others have developed methods for automatic subregional morphological assessment [48], which is a particularly valuable advancement given the spatial heterogeneity of cartilage degeneration in OA. These subregional approaches allow for the detection of focal cartilage thinning or swelling that may be masked in global measurements. In addition, DL models have been applied to automatically assess lesion severity in the cartilage, bone marrow, meniscus, and anterior cruciate ligament (ACL), capturing the complex tissue-level interactions that contribute to OA progression [47]. A recent study by Preiswerk et al. (2022) [63] used a U-Net-based DL model (a type of CNN designed for image segmentation tasks) to segment bone marrow lesion volume, achieving a Dice score of 0.70 when compared to manual segmentation. These approaches highlight the growing potential of DL to support the automatic characterization of MRI-based knee structures, potentially aiding radiologists in flagging abnormalities and monitoring OA progression. For research, these models offer scalable tools to quantify structural changes over time and enable the development of ML prediction models with MR-based predictors and outcomes.

Beyond DL-based MR image segmentation, studies have used MR imaging features as outcomes in ML-based predictive models. One recent study from Bonakdari et al. [62] (2022) assessed whether bone curvature could predict cartilage volume loss over one year in 1246 participants using five ML methods. Utilizing data from the OAI and an external Naproxen trial cohort, the authors applied and tested 5 different ML-based methods and identified the adaptive neuro-fuzzy inference system (ANFIS) (a hybrid approach combining neural networks and fuzzy logic) as the most accurate algorithm. The optimal model included five bone curvature regions (lateral tibial plateau, medial central condyle, lateral posterior condyle, and lateral and medial trochlea) enabling prediction of cartilage volume loss (12 regions), achieving high predictive accuracy (R ​≥ ​0.78), particularly in the medial condyle. This study introduces a novel, automated method for predicting cartilage loss using bone curvature predictors, validated on an external dataset.

Jamshidi et al. (2020) [61] applied ML to identify predictors of cartilage volume loss in knee OA using data from the OAI. Models included 1107 baseline features, with 135 quantitative MRI variables, and defined progression based on cartilage volume loss in the medial tibial plateau at 4 and 8 years. Progressors were those with ≥8.6 ​% (48 months) or ≥12.1 ​% (8 years) loss; non-progressors had ≤1.8 ​% or ≤1.9 ​%, respectively. Across six ML methods, key predictive features for cartilage loss included MRI-based measurements of mean cartilage thickness and surface area in the medial tibial plateau and subregions, as well as joint space width and severe medial joint space narrowing. The Multi-Layer Perceptron (MLP) (a type of feedforward artificial neural network with one or more hidden layers) achieved the highest classification performance for the 96-month outcome (AUC ​= ​0.80), while the Gradient Boosting Machine (GBM) performed best at 48 months (AUC ​= ​0.70). Together, these findings underscore the potential of MRI-based features to predict cartilage volume loss over various follow-up times.

## 6 Surgical outcomes

Total knee replacement (TKR) is a surgical procedure for knees with end-stage, radiographic OA aimed to alleviate pain, improve joint function, and enhance the overall quality of life. Therefore, predicting whether patients will advance to TKR has become an important area of research for predictive modeling. Several notable recent manuscripts are highlighted with TKR outcomes [[64], [65], [66], [67], [68], [69], [70]]; one study described below utilizes traditional ML with readily available clinical data, and another uses DL algorithms with raw images as predictors, two approaches that reflect the broader tradeoff between interpretability and performance in predictive modeling.

One recent study (Mahmoud et al., 2023) [66] using data from the OAI and MOST developed and externally validated a GBM model to predict the TKR at 2 and 5 years, achieving AUC scores of 0.91 and 0.87, respectively. Key predictors included radiographic features (e.g., KL), pain scores (e.g., WOMAC), and patient education level. The authors highlight that the ability of ML models to predict the future TKR using readily available clinical data has the potential to significantly aid in treatment planning.

Rajamohan et al. (2023) [65] developed DL models for TKR prediction over 108 months using baseline knee MRIs from the OAI. Models were trained on multiple MRI sequences using CNNs to extract features directly from image data without manual annotations. Their performance was compared to both radiograph-based DL models and a traditional model using clinical risk factors. The MRI ​+ ​radiograph ensemble model achieved the highest predictive performance (AUC ​= ​0.90), followed closely by the MRI-only ensemble model (AUC ​= ​0.89), both of which significantly outperformed the radiograph-based model (AUC ​= ​0.87) and the clinical model (AUC ​= ​0.77). External validation using 270 matched case-control pairs from the MOST study showed a modest drop in performance, with the best MRI-based models achieving AUCs between 0.66 and 0.71, underscoring the need for testing across diverse imaging protocols and populations. These findings highlight the value of MRI in DL-based TKR prediction and the potential for multi-modality models to further improve performance.

### 6.1 Emerging directions in OA prediction

Advances in OA prediction increasingly emphasize the integration of multimodal data, such as clinical features, imaging, biomechanics, and intraoperative data, to support more personalized and clinically meaningful models. ML approaches, including random forests, support vector machines, gradient boosting, and DL, have been applied across pain, radiographic, and surgical outcomes, with AutoML offering new opportunities to streamline model selection. A growing focus is on capturing individual variability in pain sensitivity through mechanisms such as inflammation, biomechanics, and psychosocial factors. In radiographic OA, attention-based deep learning and longitudinal modeling improve the ability to detect structural change, while MRI-based models leverage quantitative features such as cartilage thickness, subchondral bone, and compositional biomarkers, including cartilage T_2_ and T1rho, to detect early OA. Recently, ML models have also been applied to CT [71] and ultrasound imaging [72], which offer complementary structural information such as subchondral bone morphology and soft tissue features, expanding the range of features used in OA prediction. Additionally, ML-based phenotyping efforts have identified key imaging and biochemical markers associated with distinct progression profiles, highlighting the potential of data-driven approaches for OA prediction [73]. Explainable AI (XAI) is increasingly essential for transparency, trust, and clinical adoption of ML models in OA research; techniques such as Local Interpretable Model-Agnostic Explanations (LIME) and Shapley Additive Explanations (SHAP) provide interpretability by highlighting feature contributions, helping address key concerns around bias and transparency [74]. These developments point toward a future of precision medicine in OA, though widespread clinical adoption will require external validation, interpretability, and integration with expert decision-making.

## 7 Key findings and conclusion

This review has highlighted recent advancements in ML models for OA prediction, including clinical outcomes (pain, function), structural progression (based on radiographs and MR imaging), and risk for TKR. The Key Findings are summarized below:

• Clinical and symptom-based models: use demographic, inflammatory, biomechanical, and psychosocial factors to predict pain and functional outcomes. These models capture symptom heterogeneity and help identify phenotypes that may not be reflected in structural imaging.
• Radiograph-based models: DL models automatically grade KL and JSN, which improves standardization of radiographic evaluations, reducing inter-reader variability, and enhancing diagnostic efficiency. Predictive models for radiographic OA incidence and progression show promising performance.
• MRI-based models: Automatic quantification of features such as cartilage thickness, cartilage T_2_ relaxation time, and bone shape, including subregional assessments, supports the detection of structural changes that may not be directly evident on radiographs.
• Multimodal models: Integration of imaging and clinical data often improves the prediction of OA progression and supports personalized risk stratification.
• Translation and generalizability challenges: Despite strong internal performance, adoption of DL models remains limited by issues of interpretability, workflow integration, and lack of external validation across diverse populations and imaging protocols.

As research in the application of ML to OA prediction continues to evolve, several key considerations remain important. First, building interpretable models will be essential for clinical adoption, allowing clinicians to understand the drivers of prediction and build trust in these tools. Second, external validation using independent datasets is critical to ensure generalizability and robustness. Third, integrating multimodal data, including clinical, imaging, genetic, and biomechanical inputs, may improve predictive accuracy and support more personalized treatment strategies. A recent paper by Eckstein et al. (2025) [75] introduced the concept of multifaceted MRI, which uses multiple image contrasts in a single session to capture distinct joint pathologies such as cartilage morphometry, synovitis, and bone marrow lesions for use in ML models. This approach supports subtype-specific predictions, multi-component outcomes, and alignment of imaging biomarkers with therapeutic mechanisms in clinical trials. Another area of active development is the use of raw medical images as inputs to DL models. This fully automated approach eliminates the need for manual feature extraction and has the potential to uncover subtle patterns that may be missed by traditional methods. However, these models are generally less interpretable and require substantially larger datasets for training. In contrast, classic ML models that rely on radiologist-derived imaging features are more interpretable and data-efficient but may not capture the full richness of the imaging data. Advancements in time-series analysis will also enable better use of longitudinal data to capture dynamic changes in symptoms and function over time. Finally, the use of causal inference methods may help uncover underlying mechanisms of OA progression and point to new therapeutic targets. Taken together, these directions underscore the growing potential of ML to inform earlier detection, risk stratification, and tailored interventions in OA clinical care.

## Contributions

The authors have made substantial contributions to the following sections:

• Conception and design (GBJ CEM MCN NEL SM TML).

deep

• Drafting of the article (GBJ, TML).

• Critical revision of the article for important intellectual content (GBJ CEM MCN NEL SM TML).

• Final approval of the article (GBJ CEM MCN NEL SM TML).

## Funding source

The authors of this manuscript were funded through NIH R01-AR064771, NIH R01-AR078917, and NIH R01-AG-066474.

## Declaration of competing interest

All other authors declare no competing financial or personal interests.
